# Supplementary material for: Target-Specific Nanoparticle Polyplex Down-Regulates Mutant Kras to Prevent Pancreatic Carcinogenesis and Halt Tumor Progression
Source: Int J Mol Sci. 2023 Jan 1;24(1):752. doi: 10.3390/ijms24010752 (PMC9821664; doi:10.3390/ijms24010752)
Supplement: Supplementary file 1 [file ijms-24-00752-s001.zip › ijms-2077044-supplementary.pdf]

Supplementary Materials

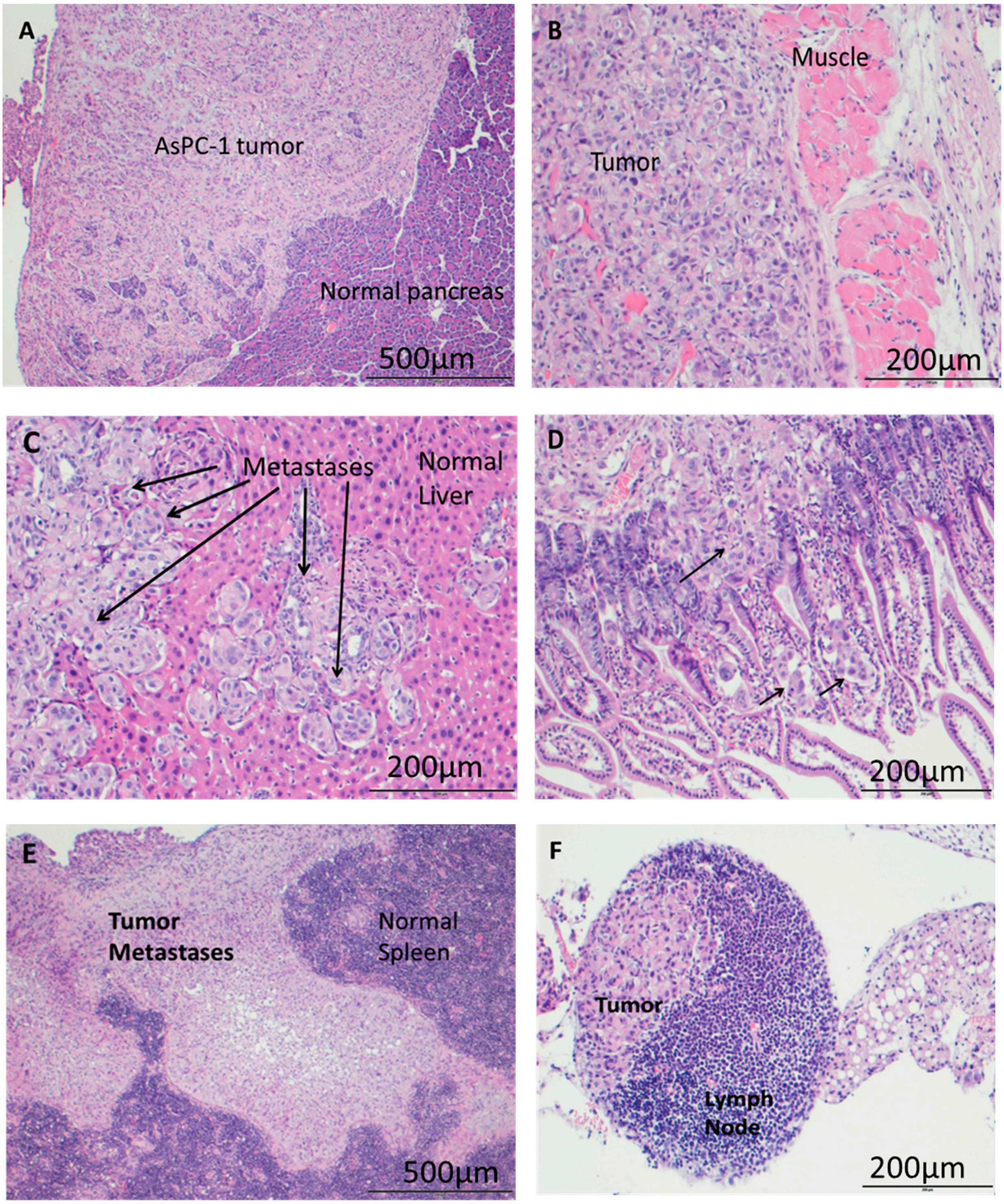

Figure S1. Confirmation of AsPC-1 tumor metastases by histology. (**A**) Primary AsPC-1 tumor in pancreas of scrambled siRNA NP-treated control mouse. (**B**) AsPC-1 tumor is shown metastasized to the abdominal wall in a *GAST* siRNA NP-treated mouse. (**C**) AsPC-1 tumor metastases confirmed in the liver

(arrows) of a mouse treated with *muKRAS* siRNA NPs. (**D**) AsPC-1 tumor metastases confirmed invading the stomach (arrows) of a mouse treated with *muKRAS* siRNA NPs. (**E**) Metastases of AsPC-1 tumor cells in the spleen of a scrambled siRNA NP treated mouse. (**F**) Lymph node invasion with the AsPC-1 tumor of a PBS-treated mouse.

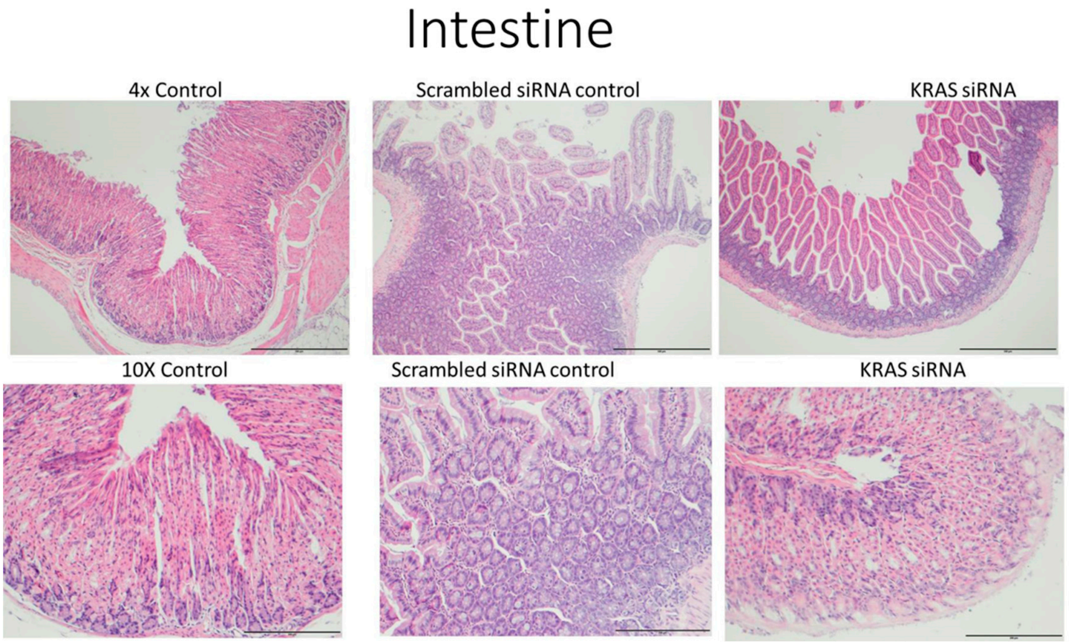

Figure S2. Mouse intestine. H&E imaged at low magnification (4X, top row; scale bar 500μm) and higher magnification (10X, bottom row; scale bar 200μm) if intestine sections from untreated (control), scrambled NP-treated, or *muKras* NP-treated revealed normal histology and no inflammation.

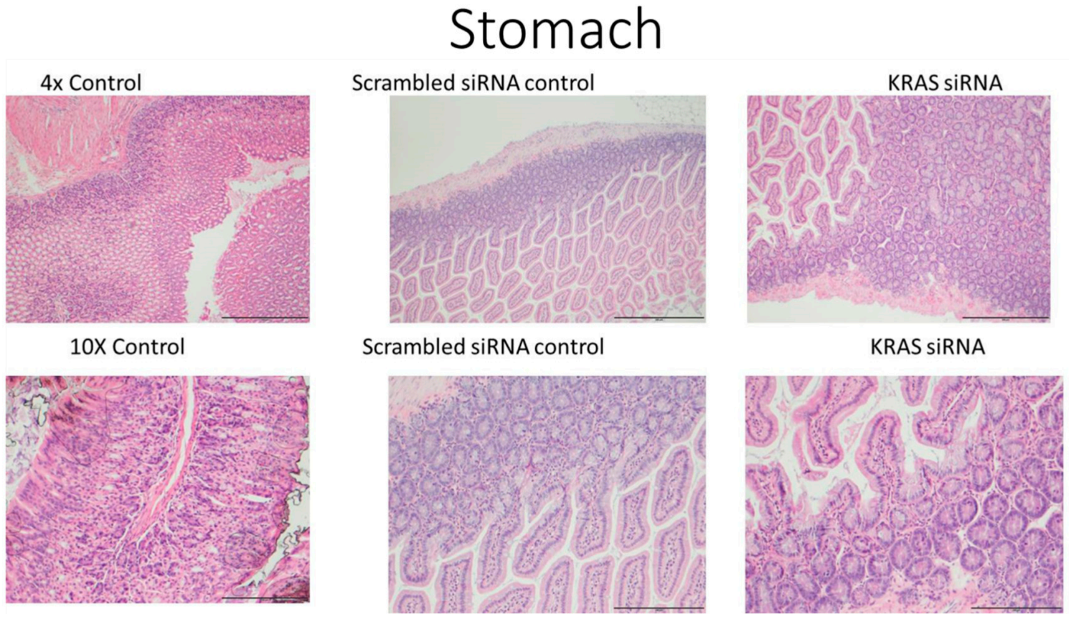

Figure S3. Mouse stomach. H&E imaged at low magnification (4X, top row; scale bar 500 μm) and higher magnification (10X, bottom row; scale bar 200μm) of stomach sections from untreated (control), scrambled NP treated, or *muKras* NP-treated revealed normal histology and no inflammation.

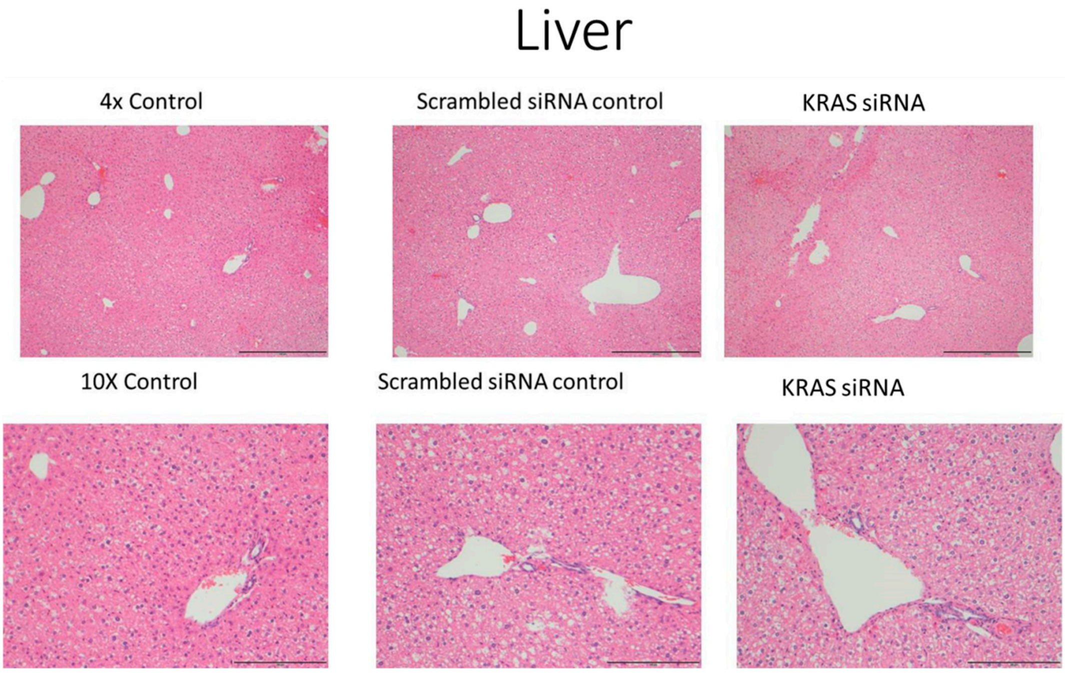

Figure S4. Mouse liver. H&E imaged at low magnification (4X, top row; scale bar 500µm) and higher magnification (10X, bottom row; scale bar 200 µm) of liver sections from untreated (control), scrambled NP treated, or *muKras* NP-treated revealed normal histology and no inflammation.

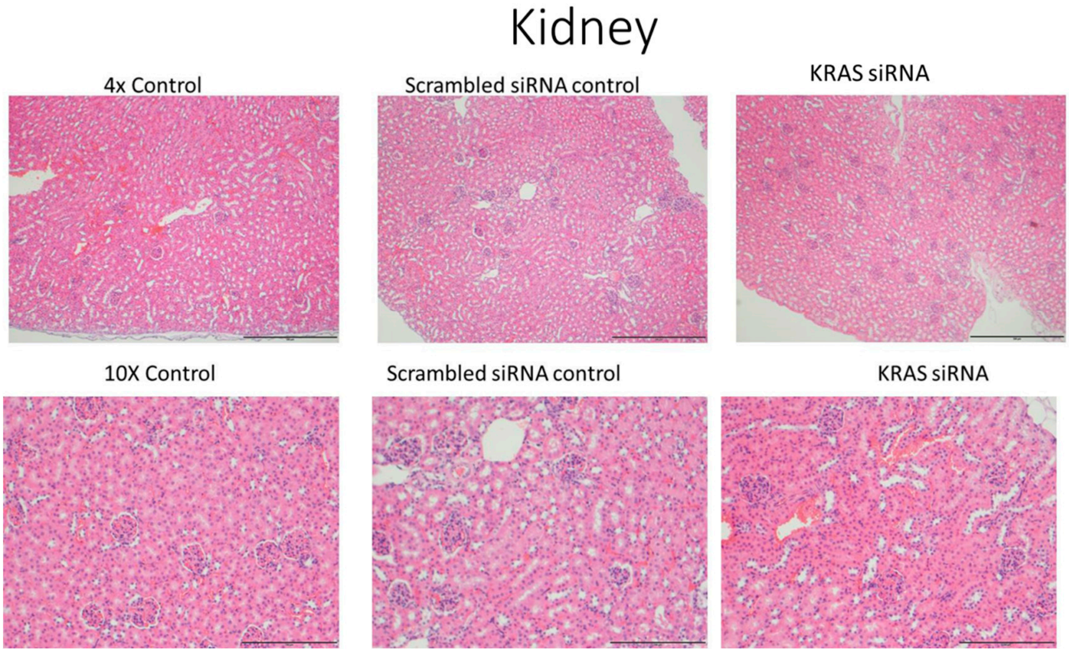

Figure S5. Mouse kidney. H&E imaged at low magnification (4X, top row; scale bar 500 µm) and higher magnification (10X, bottom row; scale bar 200 µm) of kidney sections from untreated (control), scrambled NP treated, or *muKras* NP-treated revealed normal histology and no inflammation.

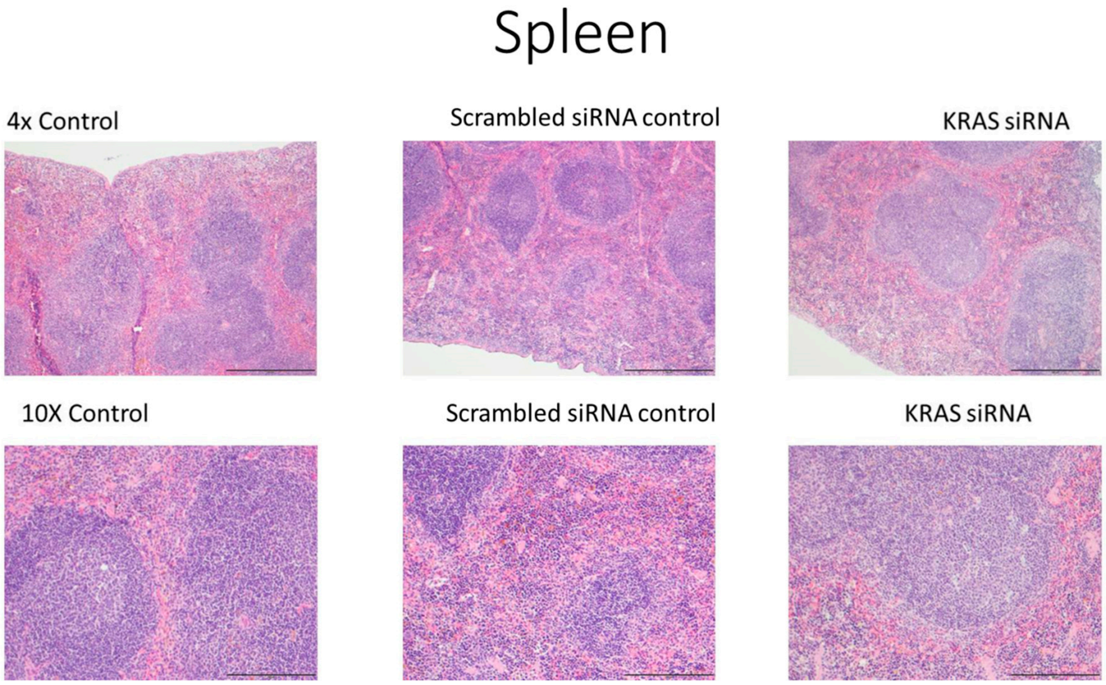

Figure S6. Mouse spleen. H&E imaged at low magnification (4X, top row; scale bar 500 μm) and higher magnification (10X, bottom row; scale bar 200 μm) of spleen sections from untreated (control), scrambled NP treated, or *muKras* NP-treated revealed normal histology and no inflammation.

Supplementary Table S1: Sequences of the siRNAs loaded to nanoparticles

siRNA	Sense 5' to 3'	Anti-sense 5' to 3'	Molecular weight, g/mol
Wild type *Kras**	GUUGGAGCU**GGU**GGCGUAGTT	CUACGCC**ACC**AGCUCCAACTT	13,447.2
Mutant, Kras */KRAS**	GUUGGAGCU**GAU**GGCGUAGTT	CUACGCC**AUC**AGCUCCAACTT	13,348.2
Scrambled Control	CGAAGUGUGUGUGUGUGGCTT	GCCACACACACACACUUCGTT	13,371.2
Gastrin (*GAST*)	GUGCUGAGGAUGAGAACUA	TAGTTCTCATCCTCAGC	11262.2

*Bold codon in the Kras sequence refers to the G12D mutation

Supplementary Table S2: Primers used for qRT-PCR.

Primer name	Forward 5’-3’	Reverse 5—3’
Mouse Mutant Kras*	**GAT**GGCGTAGGCAAGAGC	GCACGCAGACTGTAGAGCAG
Mouse Wild-type Kras	TGGTGGCGTAGGCAAGAG	GCAGACTGTAGAGCAGCGTTA
Mouse Wild-type Kras	TTGGAGCTGGTGGCGTAG	TAGAGCAGCGTTACCTCTATCG
Mouse HPRT	TCAGTCAACGGGGGACATAAA	GGGGCTGTACTGCTTAACCAG
Human Mutant KRAS	CTTGTGGTAGTTGGAGCTGATG	TGTTGGATCATATTCGTCCACAA
Human Wild-type KRAS	TGTGGTAGTTGGAGCTGGT	ATTGTTGGATCATATTCGTCCAC
Human gastrin	GCCTCTCATCATCGAAGGCA	GCCGAAGTCCATCCATCCAT
Human GAPDH	GTCTCCTCTGACTTCAACAGCG	ACCACCCTCTTGCTGTAGCCAA

*Bold codon for primer is able to differentiate wild type from mutant Kras
